# Big Data: transforming drug development and health policy decision making

**DOI:** 10.1007/s10742-016-0144-x

**Published:** 2016-03-05

**Authors:** Demissie Alemayehu, Marc L. Berger

**Affiliations:** Pfizer Inc., 235 East 42nd Street, New York, NY 10017 USA

**Keywords:** Keywords Big Data, Drug development, Predictive analytics, Real world data, Personalized medicine

## Abstract

The explosion of data sources, accompanied by the evolution of technology and analytical techniques, has created considerable challenges and opportunities for drug development and healthcare resource utilization. We present a systematic overview these phenomena, and suggest measures to be taken for effective integration of the new developments in the traditional medical research paradigm and health policy decision making. Special attention is paid to pertinent issues in emerging areas, including rare disease drug development, personalized medicine, Comparative Effectiveness Research, and privacy and confidentiality concerns.

## Introduction

The rapid increase in the quantity, diversity and accessibility of digitized patient data has presented unprecedented challenges and opportunities for drug development, regulatory reviews, and healthcare utilization and decision making (Mayer-Schönberger and Cukier 2014; Roski et al. 2014). In contrast to the existing paradigm of drug development that relies on systematically collected numeric data, the new reality involves information that comes in diverse forms and shapes. In this context, Big Data means not only electronic health records, claims data but also data captured through every conceivable medium, including Social Media, Internet search, wearable devices, video streams, and personal genomic services; it may also include data collected from randomized controlled clinical trials (RCTs), particularly when dealing with high dimensional data, including genomic, laboratory, or imaging data.

Arguably, one of the most promising aspects of Big Data in the healthcare arena is its budding role in promoting and advancing research in personalized and precision medicine (Panahiazar et al. 2014; Teli 2014). At the operational level, there is also a significant function for Big Data to enhance the design and conduct of clinical trials, ranging from refining design parameters to identifying patients likely to benefit from experimental medicines. In rare disease research, the accessibility of additional data may have the added advantage of helping fill the gap created by the widely recognized paucity of information (Clarke et al. 2011). Further, there are discernibly important implications for Comparative Effectiveness Research (CER), where the growing need to establish the relative risks and benefits of alternative medical interventions requires evidence base beyond that can be provided by conventional RCTs (Berger and Doban 2014; Gray and Thorpe 2015).

The accompanying developments in methodological procedures and data visualization can also help to improve operational efficiency in the execution of trials, and to tackle complex analytical issues that cannot readily be dealt with using traditional approaches. The potential of these developments to contribute to efforts to reduce costs and to accelerate the delivery of drugs to patients that need them is immeasurable (LaValle et al. 2011).

On the other hand, Big Data in turn poses considerable technical, analytical and ethical challenges. In the face of vast amounts of data, the traditional approaches that rely on transactional database management systems may no longer be satisfactory to link, integrate and process the heterogeneous data emanating from disparate sources *(*Hilbert and López 2011). The unprecedented volume of information also needs new computational software and hardware capabilities (Assuncao et al. 2013). Analytically, most traditional approaches break down in the face of highly dimensional data (National Research Council 2013). Furthermore, uncritical use of modern algorithmic tools is likely to lead to unacceptable results with unpredictable consequences (Lazer et al. 2014).

Over and above the technical and analytical challenges, there are also the lingering issues of privacy and confidentiality, and whether the data is good enough to support health policy decision-making (Fhom et al. 2015). Concerning privacy and confidentiality, much work is needed in terms of formulating guidelines to help drug developers understand the current thinking about the extent and nature of evidence from Big Data that would be deemed admissible in the drug approval process (Federal Trade Commission 2010; European Parliament, Council of the European Union 1995). A rationale and pragmatic approach entails a firm understanding of the balance between the data need for medical research and the protection of patient privacy.

With respect to the quality of the data, there is a vibrant debate in the scientific community regarding whether real world data is of sufficient quality for evidence-based medicine. If it’s not, then many believe that the issue of GIGO (e.g., garbage in–garbage out) applies. Many others, including ourselves, argue that although much of real world data is sparse and a lot of the data is “dirty”, with proper analytical, computational and data management tools, it is still useful and can support health policy decision-making.

In this paper, we provide a high-level overview of the challenges and opportunities of Big Data vis-à-vis drug development, with emphasis on the potential for transforming the current paradigm of clinical research and regulatory review, advancing personalized medicine, and protection of the privacy of study participants. The paper is organized as follows. In Sect. 2 we highlight the place of Big Data in evidence-based medicine, including research in rare diseases and personalized medicine. In Sect. 3 we review the implication of the development of new analytical tools in addressing lingering issues in medical research. In the rest of the paper we discuss some of the challenges in incorporating Big Data in clinical development and conclude with suggested recommendations for further work.

## Big Data in drug development and healthcare policy

### Transforming the drug development paradigm

Incontrovertibly, randomized controlled trials (RCTs) are considered the “gold standard” for generating evidence relating to the comparative risks and benefits of alternative treatment options. Indeed, they hold a prominent place in the hierarchy of evidence, principally because of the role of randomization in ensuring internal validity, ruling out the effects of potential confounding factors (Barton Barton 2000). However, RCTs also have their own limitations. First and foremost, they lack external validity, since the circumstances under which they are executed may not be reflective of real-world experience. Further, for operational or ethical reasons, it may not even be feasible to conduct them. Consequently, there is a growing interest in complementing or buttressing evidence from RCTs using data from other sources, including observational studies, claims and electronic health record (EHR) databases, or other nonstandard and nonconventional information sources.

In the context of the tradition drug development paradigm, the availability of data from observational studies or other sources can seamlessly be integrated in the various phases of clinical research, ranging from supporting proof of concept (PoC) study designs to assessing the safety profile of an approved drug in a post-marketing setting.

In early phase trials, where information needed to characterize the safety and efficacy of a new medicine may often be deficient, data from secondary sources may help to define endpoints, characterize study populations and shed light on relevant aspects of other relevant design parameters. For example, for studies designed in a Bayesian framework, informative priors for a placebo or a well-established control group may be determined from observational data to gain efficiencies (Walley et al. 2015).

This is particularly germane to rare disease research, which is characterized by the small size of the target population, and poorly understood natural history of the disease (Augustine et al. 2013). In many cases the diseases are life-threatening and may not even have any drug development precedent. Data from observational studies can positively help mitigate some of these problems, including development of outcome assessment tools, characterization of disease presentation and disease progression, and better understanding of natural histories.

### Role in pharmacovigilance

In the recent literature, much attention has been paid to the role of data from secondary sources in the characterization of the risk and benefit of drugs. Since RCTs used for registration purposes are typically underpowered to reveal safety signals for rare events, there is a lot of focus on evidence generated based on observational data (Finkle et al. 2014). Indeed, the FDA Sentinel Initiative relies on a distributed real world data network (Robb et al. 2012). Using data drawn from queries entered into Internet search engines, evidence of unreported prescription side-effects were detectable before they were found by the FDA’s warning system (White et al. 2013). Moreover, Big Data mining has also been reported to reveal safety signals that conventional pharmacovigilance approaches were unable to detect (Gooden et al. 2013).

### Advancing personalized medicine

While the use of observational data has mostly been limited to population studies, the potential of Big Data in precision medicine is yet to be fully realized. In other industries, much progress has been made to utilize Internet search data and modern predictive analytics techniques to profile customers for the purpose of targeting and enticing them to promote business. The so-called ‘anticipatory shipping’ patent filed by Amazon is a case in point (Kopalle 2014). The idea can certainly be extended to advance personalized medicine. Huge quantities of information may be accessed from personal genome services, which provide direct-to-consumer genetic testing and aggregated customer data for use in medical research (Genetics and Public Policy Center 2009). Wearable devices, which can continuously monitor activity and health state, can provide data in real-time about an individual, which can in turn be used in early diagnosis of diseases and to personalize treatment (Zheng et al. 2013).

### Comparative effectiveness research

In CER, Big Data is poised to play a critical role, providing the opportunity to analyze diverse digitized health data to make decisions about the relative benefits of drugs, and their use in the real-world setting (Gray and Thorpe 2015). Notably, evidence from observational studies may help to fill information gap for which there is inadequate data from RCTs (Berger et al. 2015). Currently the FDA is sponsoring an initiative through the Brookings Institute to examine how “evidence from clinical experience (ECE)” can inform regulatory decision-making. One important issue that needs to be addressed is that the effect sizes in many comparative effectiveness studies are relatively small, hence there is skepticism regarding the robustness of such findings in a regulatory context. Another important issue is whether one can infer causality, rather than just correlation, from observational studies, as will be further expounded below. Further, there needs to be increased focus on a thorough understanding of the operating characteristics of commonly used procedures.

### Informing quality improvement efforts and a learning health care system

Large providers and insurers are routinely mining their data to examine costs and health outcomes. The insights from these analyses inform their reporting on quality of care indicators as well as their design of care and case management programs. The current focus of much of this activity is to develop better predictive models to assess progression of disease and attendant health care resource use, as well as the response to therapy. Such efforts will provide the engine for a learning health care system (McElwee and Dubois 2015).

With the rising cost of conducting clinical trials, there has been increased focus on data-driven approaches to help improve the quality of data. Non-profit organizations, such as TransCelerate Biopharma and regulatory agencies (FDA 2013; EMA 2013), have focused on a risk-based monitoring framework, which can be informed by real world data on distribution and characteristics of patients, as well as investigators and other site performance metrics. Accordingly, real world data is being used to refine design parameters including definition of inclusion/exclusion criteria, honing inputs for sample size determination, and development of simulated trials to proactively identify and address potential issues that arise in the course of the trial conduct.

### Informing health technology assessments

Real world data generated at the point of care, including claims data of increasing quality, can be leveraged to make critical decisions regarding access to and pricing of new therapies, by both payers and drug developers. Such data can help characterize diseases and patient populations, evaluate treatment patterns and adherence, gain information on competitors, and target underserved patient groups (Garrison et al. 2007). Further, because of the recognized inadequacy of RCT data used for registration to infer about the experience of patients in real-life setting, there is a growing demand for real world evidence by regulatory authorities, payers and healthcare providers to fully evaluate the cost-benefit of a new treatment (Akhmetov 2015). In the context of the reimbursement of personalized medicine, Big Data will play even a more critical role in addressing some of the challenges faced by payers and drug makers (Faulkner et al. 2012).

### Informing shared decision-making at the bedside

In the future, one can imagine that a practitioner will have real-time access to a database that will allow her to rapidly analyze how subsets of patients similar to the one in the examination room (e.g., demographically, principal health problem, general health status, etc.) have been treated and what were their outcomes. This will inform shared decision making by patients and their providers. However, the use of rapid-cycle analytics at the bedside remains controversial today. Nonetheless, in the absence of good insights from the published clinical trials, providers will increasingly seek them from accessible real world data bases (Greenwood et al. 2014).

## Methodological reflections

### Extenuating bias and confounding

Historically, a major methodological issue with observational studies in generating evidence has been the handling of bias that arises in the absence of randomization. This has often been tackled using suitable design and/or analytical strategies. Depending on the objective of the study and data sources, various design options are available to mitigate bias, including cohort, case–control and self-controlled case-series approaches. In addition, pragmatic studies, which mimic real-world drug use, while incorporating randomization, are increasingly used to mitigate bias. Analytically, methods such as propensity score and regression analyses have been used to handle observed confounders, while such techniques as instrumental variables are frequently implemented for latent confounders (Berger et al. 2009, 2012). Nonetheless, recent studies have shown that results based on existing approaches are quite dependent on database and methodological choices, and that the accompanying *p*-values and confidence levels are unreliable, since they are based on incorrect assumptions about underlying null distributions (Madigan et al. 2013). Although modern algorithmic techniques are now brought to bear to mitigate the shortcomings of the conventional approaches, including high dimensionality (Lee et al. 2010), caution is required in the interpretation of the accompanying results in view of their sensitivity to database and methodological choices.

### Handling nonstandard data

Along with the approaches pertaining to the minimization of bias discussed above, there are other methodological developments that have considerable import in the use of observational data in medical research. Classification and regression trees, including recent enhancements such as random forests, are routinely used in subgroup identification (Foster et al. 2011; Breiman 2001). Regularized regression procedures are now widely used to handle highly dimensional data, particularly in genomics (Li et al. 2007). Alternative data mining tools are available to characterize the safety profiles of drugs (Harpaz et al. 2012). Object oriented data analytic techniques make it possible to tackle data from nom-Euclidean manifolds (Wang and Marron 2007). The so-called topological data analysis (TDA) has been applied to understand patterns in data-driven exploratory settings (Nielson et al. 2015). Traditional methods for detecting outliers are also enhanced to handle high-dimensional cases (Vu and Gopalkrishnan 2010).

These new methodological frontiers have also brought with them challenges of their own, particularly their opacity and the inaccessibility of their operating characteristics to the general audience. Uncritical application of the methods may also lead to unreliable results with untoward consequences for public health (National Research Council 2013).

An additional issue is the challenge of linking data from multiple sources, some of which may be linked and some not. This is not just a technical issue of linkage, but concerns data definitions and how data models are designed. It has been reported that the use of different common data models can impact the results of analyses (Xu et al. 2015).

### Opportunity for interdisciplinary collaboration

The generation of actionable knowledge from the vast and complex data generated through high-throughput technologies in genomic and other medical research, as well as data collected via healthcare providers, wearable devices and other electronic media requires the collective expertise of researchers from diverse disciplines. This has in particular presented considerable opportunity for collaborative efforts among statisticians and computer scientists. For example, core capabilities from the two disciplines are leveraged to tackle some of the major Big Data problems, including communication and privacy, in the framework of statistical risk minimization, while taking advantage of the computational capabilities that traditionally exist in the computer science domain (Duchi et al. 2013).

### Causation versus association

With the advances made in the so-called algorithmic approaches to data analysis, there is always the danger of over interpreting results and ignoring fundamental issues, such as data interdependencies and multiplicity problems. As articulated in a recent article in Science “…quantity of data does not mean that one can ignore foundational issues of measurement and construct validity and reliability and dependencies among data” (Lazer et al. 2014). Indeed, to maximize the value of Big Data in advancing medical science, one should refrain from the uncritical application of readily available algorithmic techniques, and use caution in interpreting results, in the search for patterns and associations in massive data sets. While there is a general consensus about the use of available methods for hypothesis generation, it is prudent to remain skeptical regarding the inference of causation. One of us has argued that rigorous well-designed and well-executed observational study can suggest actionable evidence of causal relationships (Berger et al. 2012).

## Challenges with incorporation of RWD in drug approval

### Technical barriers

The growth in volume and complexity of data has required new technological solutions to facilitate the accessibility and linkage of information from the different sources (Hilbert and López 2011). The volume and variety of data necessitates developing highly distributed architectures, introducing increased memory and processing power, and leveraging open-source licensing options (Assuncao et al. 2013). Unlike traditional relational database systems, new platforms, such as Apache Hadoop, are needed to manage unstructured data, as well as data of diverse formats. Cloud solutions with High Performance Computing (HPC) are increasingly relied upon for tasks that traditional computing facilities cannot handle.

Despite the promise of wearable devices to provide real-time data on the health status of individuals, there are still outstanding issues of harmonizing the information gathered from diverse device types. In addition, there is presently no coherent effort to validate the various devices in popular use, or to create a framework to dependably store the data for aggregation purposes.

### Analytical issues

Despite the considerable advances made in Big data analytics, there are still pertinent methodological issues that limit the potential use of the so-called machine learning tools in evidence-based medicine. In most cases, the operating characteristics of the procedures are not fully explored, and typical applications tend to focus on hypothesis generation, rather than confirmation. Indeed, the introduction of such tools as false discovery rates (FDR) notwithstanding, the issue of multiplicity remains pervasive.

For historical reasons, the development of most of the widely used techniques has evolved in silos, with little or no collaboration among key stakeholders. As was acknowledged in a recent report (National Research Council 2013), there now appears to be a realization that “… massive data analysis is not the province of any one field, but is rather a thoroughly interdisciplinary enterprise. Solutions to massive data problems will require an intimate blending of ideas from computer science and statistics, with essential contributions also needed from applied and pure mathematics, from optimization theory, and from various engineering areas, notably signal processing and information theory.”

### Ethical concerns

An equally important challenge is the ethical issue about ownership of the data. At present there is no clear regulatory or legal framework or guidelines for use of Big Data in advancing medical research (see, e.g., Gray and Thorpe 2015; Williams and Javitt 2006). This requires the collaborative efforts of various stakeholders, including legal scholars, sociologists, and other pertinent professionals. Some of the steps that need to be taken may require enhancing existing measures to regulate the collection and use of health information, with particular emphasis on the challenges posed by the explosion of digitized data outside of the traditionally recognized healthcare sector. A critical component of a viable policy should also be the recognition of the role to be played by patients in the decision making regarding the use of personal information to advance medical science. Most importantly, a responsible public policy should be one that does foster innovation, while protecting privacy and the confidentiality of personal data.

### Regulatory framework

From drug licensing perspectives, there is no definitive standard about the acceptability of data from non-RCT sources to support approval of a new medicine for use in humans. Hitherto, use of observational data has mainly been limited to the assessment of safety signals, and, in frequently, in post hoc exploration of drug utilization and other cost-benefit evaluations. In certain situations, including rare disease research, it may be essential to rely on data available at the time of drug approval (see, e.g., Stuart et al. 2001, for a research direction pertaining to the generalizability of results from RCTs). While the recent passage of the “21^st^ Century Cures Act” in US congress may eventually pave the way for use of data from other sources to support approval of new drugs (H.R.6 - 21st Century Cures Act 2015), more effort should be exerted to formulate a clear policy about the value and locus of such data in the evidence generation continuum for drug development and licensing. As mentioned earlier, there is an FDA sponsored initiative underway examining this issue.

## Conclusion

By all accounts, the digital data era is poised to impact and revolutionize the development and targeting of new medicines. As real-world data becomes increasingly ubiquitous, it will routinely be used in healthcare decision-making and in providing actionable insights. However, to optimize the leveraging of these data, it is critical to understand the underlying limitations and associated challenges, and put in place mitigating measures.

A critical success factor for effective use of digitized data in drug development is a robust infrastructure that accommodates the volume, diversity and speed of the information generated by disparate sources and media. This should, of course, be accompanied by complementary methodological developments that seamlessly combine the elegance of traditional statistical theory, with the computational efficiencies honed in computer science and related domains. Such a task would indubitably require genuine collaborative efforts among pertinent disciplines, including statisticians, computer scientists and software engineers. In addition, there should be a concerted effort to recognize the underlying issues with disparate data generated by different owners, who may not have consistent agendas, and put in place an effective and transparent framework that would accelerate the use of data to advance medical research.

In the new era of digitized data, the need to protect patient privacy and confidentiality is more imperative than ever before. New ethical standards are required to ensure that information from individual subjects is properly used to advance medical science and to develop cures for hard-to-treat diseases. A balanced approach to protecting privacy, while promoting science, entails the concerted efforts of all relevant players, including ethicists, medical professionals, legal experts, and other stakeholders.

While there are promising signals in the regulatory arena, much work is still needed to give drug developers the requisite guidance for use of Big Data in supporting New Drug Applications (NDAs). Current guidelines are either limited to post-marketing safety surveillance or drugs intended for rare diseases. The recent announcement by the European Medicines Agency (EMA) to launch the so-called “adaptive licensing pilot project” has the implicit intent of encouraging sponsors to use data from real-world experience to support approval for gradual use by broader patient populations (EMA 2014). In the United States, the implementation of the 21st Century Cures Act may promote the development of concrete guidelines on the use of data from patient experience to support NDAs (H.R.6 - 21st Century Cures Act 2015).
